# B cell–activating factor modulates the factor VIII immune response in hemophilia A

**DOI:** 10.1172/JCI142906

**Published:** 2021-04-15

**Authors:** Bhavya S. Doshi, Jyoti Rana, Giancarlo Castaman, Mostafa A. Shaheen, Radoslaw Kaczmarek, John S.S. Butterfield, Shannon L. Meeks, Cindy Leissinger, Moanaro Biswas, Valder R. Arruda

**Affiliations:** 1 Department of Pediatrics, Perelman School of Medicine, University of Pennsylvania, Philadelphia, Pennsylvania, USA.; 2 Divison of Hematology, Children’s Hospital of Philadelphia, Philadelphia, Pennsylvania, USA.; 3 Herman B. Wells Center for Pediatric Research, Indiana University School of Medicine, Indianapolis, Indiana, USA.; 4 Center for Bleeding Disorders and Coagulation, Careggi University Hospital, Florence, Italy.; 5 Department of Pediatrics, Aflac Cancer Center and Blood Disorders Center at Children’s Healthcare of Atlanta, Emory University School of Medicine, Atlanta, Georgia, USA.; 6 Section of Hematology/Oncology, Tulane University School of Medicine, New Orleans, Louisiana, USA.; 7 Raymond G. Perelman Center for Cellular and Molecular Therapies, Children’s Hospital of Philadelphia, Philadelphia, Pennsylvania, USA.

**Keywords:** Hematology, Coagulation, Cytokines

## Abstract

Inhibitors of factor VIII (FVIII) remain the most challenging complication of FVIII protein replacement therapy in hemophilia A (HA). Understanding the mechanisms that guide FVIII-specific B cell development could help identify therapeutic targets. The B cell–activating factor (BAFF) cytokine family is a key regulator of B cell differentiation in normal homeostasis and immune disorders. Thus, we used patient samples and mouse models to investigate the potential role of BAFF in modulating FVIII inhibitors. BAFF levels were elevated in pediatric and adult HA inhibitor patients and decreased to levels similar to those of noninhibitor controls after successful immune tolerance induction (ITI). Moreover, elevations in BAFF levels were seen in patients who failed to achieve FVIII tolerance with anti-CD20 antibody–mediated B cell depletion. In naive HA mice, prophylactic anti-BAFF antibody therapy prior to FVIII immunization prevented inhibitor formation and this tolerance was maintained despite FVIII exposure after immune reconstitution. In preimmunized HA mice, combination therapy with anti-CD20 and anti-BAFF antibodies dramatically reduced FVIII inhibitors via inhibition of FVIII-specific plasma cells. Our data suggest that BAFF may regulate the generation and maintenance of FVIII inhibitors and/or anti-FVIII B cells. Finally, anti-CD20/anti-BAFF combination therapy may be clinically useful for ITI.

## Graphical abstract

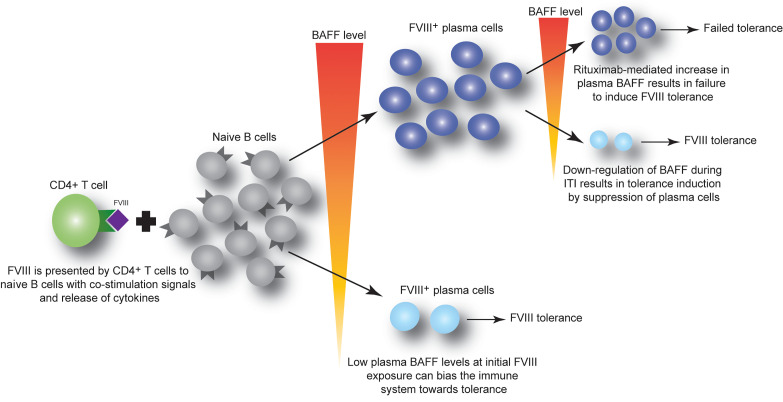

## Introduction

One of the most immunogenic protein-based therapies is coagulation factor VIII (FVIII) (1), which is missing or defective in the X-linked congenital bleeding disorder hemophilia A (HA). HA results from mutations in the *F8* gene and affects 1 in 5000 males born worldwide (2). Prophylactic FVIII replacement therapy prevents bleeding-related morbidity and mortality (3), but the formation of anti-FVIII (α-FVIII) neutralizing alloantibodies, termed inhibitors, represents the most significant therapeutic complication.

High-titer inhibitors occur in approximately 20% to 30% of patients with severe HA (<1% residual FVIII activity), generally at a young age and within the first 50 exposure days to FVIII products (4). Inhibitor titers are measured in Bethesda units (BU), where 1 BU is equal to the amount of antibody that neutralizes 50% of FVIII activity (5). High-titer inhibitors (BU > 5) render replacement therapy ineffective and result in increased morbidity and mortality (6–9). Although the bispecific antibody emicizumab, which mimics FVIII activity, is useful for prophylaxis against bleeding, patients still require additional treatment with bypassing agents when inhibitors are present for breakthrough bleeds and/or surgery (10–12). Thus, the prevention and/or eradication of inhibitors of FVIII is of fundamental interest in the optimal management of HA (13). Several genetic and environmental risk factors have been implicated in inhibitor formation (14), including *F8* mutations (15); however, there is no clear predictor for which patient will go on with certainty to develop an antibody response.

A protracted course of high-dose FVIII infusions, termed the immune tolerance induction (ITI) protocol, is the only widely accepted method for establishing FVIII immunologic tolerance (16). As inhibitors typically develop at a median age of 15 months, ITI usually requires placement of a central venous catheter, which poses thrombotic and infectious risks (17). Although ITI is successful in 60% to 70% of selected patients with “good” risk factors (18), there is a 5% to 35% recurrence risk (19), with higher rates seen in patients who required addition of immunosuppressive agents after failing initial ITI (20). A variety of immunomodulatory drugs have been tried in inhibitor patients with varying success rates and toxicities (21–24). Rituximab, a chimeric α-CD20 mAb that depletes mature B cells, has been tried for inhibitor eradication in ITI-refractory patients. However, both a prospective trial of rituximab alone and a retrospective review of rituximab use with FVIII ITI in HA patients demonstrated limited success at tolerance induction (25, 26). The reason for this modest effect remains unclear, and optimization of this therapeutic strategy with adjuncts to rituximab have not been pursued so far.

The basic mechanisms driving FVIII inhibitor development remain incompletely understood. Current evidence, predominantly from animal studies, suggests that exogenous FVIII is taken up in the spleen by marginal zone (MZ) B cells, MZ macrophages, or delivered to other antigen-presenting cells (APCs) and presented via major histocompatibility (MHC) type II molecules to CD4^+^ T cells (27–31). Under conditions of costimulation, CD4^+^ T cells, in particular T follicular helper (Tfh) cells, activate cognate B cells to mature and proliferate within germinal centers (GCs) into antibody-secreting plasma cells (PCs) or memory (MEM) B cells (32, 33). Long-lived PCs (LLPCs) ultimately settle in the bone marrow (BM), sustaining the humoral response for extended periods. In humans, the antibody response against FVIII consists of both neutralizing (high affinity, IgG_4_) and nonneutralizing (low affinity, IgG_1_) antibodies (34), likely from B cell receptor (BCR) rearrangement driving affinity maturation. In mice, it is generally thought that IgG_1_ most closely mimics human IgG_4_ (35), but inhibitory α-FVIII antibodies of many subclasses have been described (36, 37). Prior studies have implicated either plasma levels of cytokines that mediate B and T cell cross-talk, such as IL-2, IL-10, and TNF-α (38), or SNPs in regulatory elements of these genes in the FVIII immune response. However, B cell–specific cytokines have not been rigorously investigated previously, save one report (39).

A particular TNF family of cytokines and receptors has been implicated in the survival and maturation of B cells (40). This family includes the soluble ligands B cell–activating factor (BAFF, also known as BLyS) and a proliferation-inducing ligand (APRIL) as well as their receptors BAFF-R (BR3), transmembrane activator and calcium modulator and cytophilin ligand interactor (TACI), and B cell maturation antigen (BCMA), the latter of which can be shed from the cell surface by γ-secretase and thus measured in plasma (41). BAFF binds with greatest affinity to BAFF-R (42) and downstream signaling from this interaction via the noncanonical NF-κB and PI3K signaling pathways supports the survival and maturation of transitional (TR), MZ, and other peripheral B cell subsets (40). APRIL binds to TACI and BCMA to promote PC differentiation and survival. Expression of both BAFF and APRIL is known to be increased under proinflammatory conditions (43), thus contributing to pathogen responses (44).

Elevations in plasma BAFF levels have been implicated in several autoimmune disease processes, leading to the development of a clinically approved α-BAFF mAb, belimumab (45–48). Recent studies in allograft transplant recipients demonstrate that high BAFF levels prior to transplant are associated with antibody-mediated rejection and elevated levels following α-CD20 therapy may also contribute to the failure to induce humoral tolerance to the graft (49–51). Using both adult and pediatric HA patient samples and HA mouse models, here we investigate the hypothesis that BAFF may play a role in the generation and sustenance of α-FVIII antibodies, especially in the context of α-CD20 therapy, which may influence therapeutic efficacy. These findings may offer additional therapeutic targets for FVIII inhibitors in HA.

## Results

### Analysis of samples from HA patient cohorts of distinct ages and geographic backgrounds

#### BAFF levels are higher in pediatric HA patients with persistent FVIII inhibitors and correlate with α-FVIII antibody titers.

**Figure 1:**
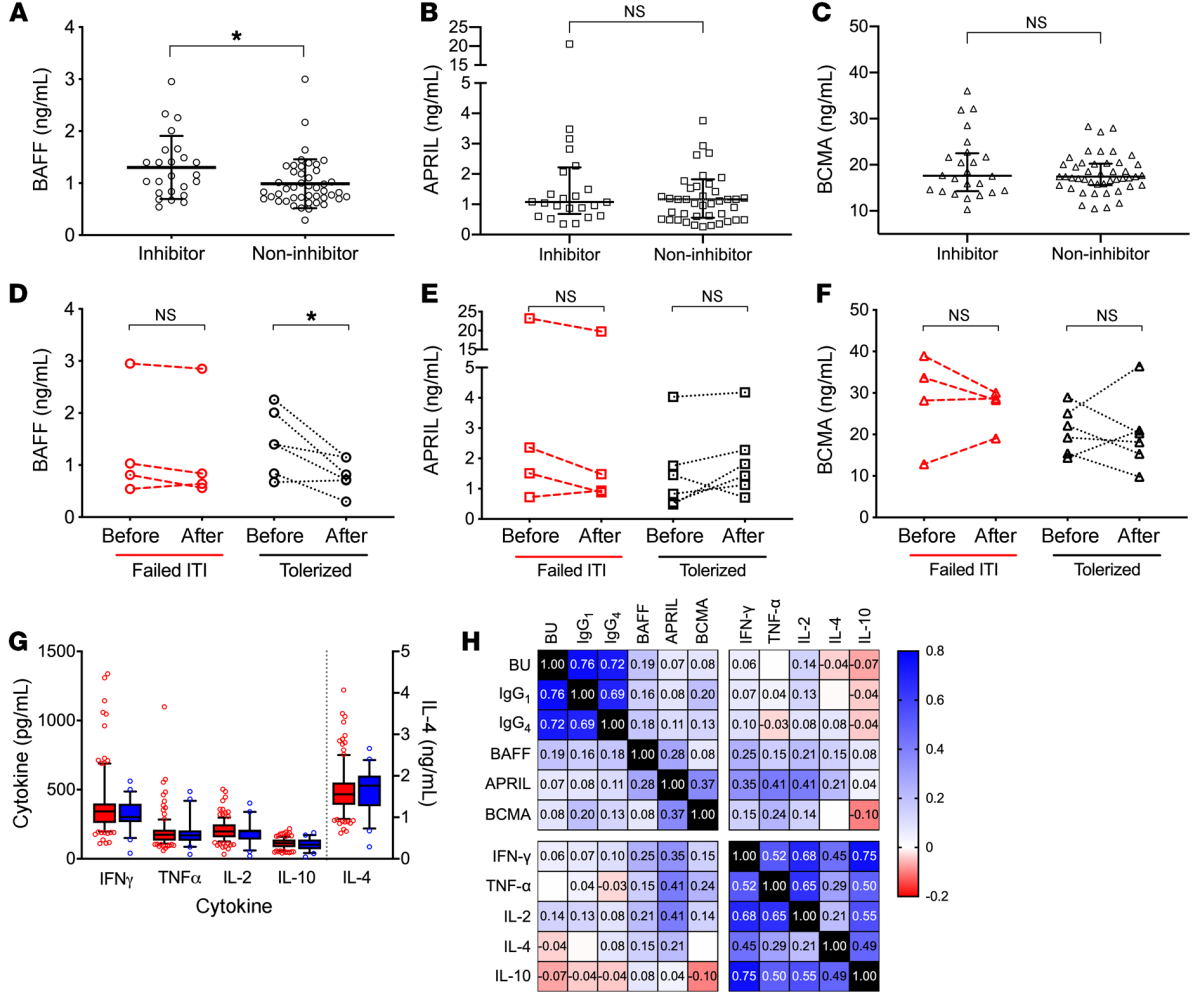
B cell cytokine levels in pediatric patients with hemophilia A. B cell cytokines in pediatric HA patients with FVIII inhibitors (*n* = 24) or without FVIII inhibitors (*n* = 45). (**A**) BAFF levels. (**B**) APRIL levels. (**C**) BCMA levels via unpaired *t* test. Longitudinal analysis of (**D**) BAFF, (**E**) APRIL, and (**F**) BCMA levels in pediatric patients with inhibitors who failed immune tolerance induction (*n* = 4) or succeeded (*n* = 6) via paired *t* test. (**G**) Peripheral T-helper cytokine levels in pediatric HA patients with (red squares) and without (blue squares) inhibitors via unpaired *t* test. (**H**) Heatmap of Spearman’s correlation of Bethesda titer, α-FVIII IgG subclasses, and cytokines. Box-and-whisker plots show median with 25%–75% IQR, whiskers delineate 10th and 90th percentiles, with values outside these ranges shown as symbols. Other data plotted as mean ± SD. *P < 0.05. NS, not significant.

Plasma samples from 69 patients followed at the Children’s Hospital of Philadelphia (CHOP) Hemophilia Treatment Center (HTC) were collected longitudinally. Demographic and baseline characteristic data for these patients are summarized in Table 1. Of the 69 pediatric patients, 24 (34.8%) had FVIII inhibitors and 45 (65.2%) did not. Patient age did not differ between those with inhibitors (median 3.41, IQR 1.79–7.71 years) versus noninhibitor controls (median 8.08, IQR 1.79–13.50 years; *P* > 0.05 by Mann-Whitney *U* test). Consistent with prior studies (52, 53), patients with inhibitors were more likely to have *F8* gene inversions or large deletion mutations compared with noninhibitor patients (58.3% vs. 26.7%, *P* < 0.05). There was no difference in race or disease severity among HA patients with and without inhibitors. All patients were treated with recombinant FVIII products. Only 2 of 69 patients were female and neither had FVIII inhibitors; both have a normal karyotype with *F8* intron 22 inversion mutations and are presumed to have severe, skewed X-chromosome inactivation. Plasma BAFF levels (Figure 1A) were higher in HA patients with inhibitors compared with those without inhibitors (1.30 ± 0.61 vs. 0.99 ± 0.47 ng/mL, *P* = 0.021 by *t* test). Moreover, BAFF levels decreased from baseline in HA inhibitor patients who underwent ITI and achieved FVIII tolerance from 1.43 ± 0.63 to 0.81 ± 0.32 ng/mL (paired *t* test *P* = 0.025, Figure 1D). In comparison, those who failed ITI had steady levels at 1.33 to 1.23 ng/mL (paired *t* test *P* = 0.246, Figure 1D). Levels of APRIL (2.55 ± 4.66 vs. 2.97 ± 7.49 ng/mL, *P* > 0.05) and BCMA (20.09 ± 6.99 vs. 17.99 ± 4.08 ng/mL, *P* > 0.05) were not different between pediatric HA patients with and without inhibitors (Figure 1, B and C), or between those who achieved and failed to achieve FVIII tolerance (Figure 1, E and F).

Levels of the T-helper cytokines IFN-γ, TNF-α, IL-2, IL-4, and IL-10 did not differ between pediatric HA patients with and without inhibitors (Figure 1G). BAFF levels correlated with α-FVIII IgG_1_ (Spearman’s correlation coefficient [*ρ*] = 0.16, *P* < 0.05) and α-FVIII IgG_4_ (Spearman’s *ρ* = 0.18, *P* < 0.01) and most strongly with the α-FVIII Bethesda titer (Spearman’s *ρ* = 0.19, *P <* 0.005). Correlation plots are shown in Supplemental Figure 1 (supplemental material available online with this article; https://doi.org/10.1172/JCI142906DS1). In contrast, none of the other cytokines correlated with Bethesda titer or IgG subclasses, with Spearman’s *ρ* ranging from –0.03 to 0.14 (*P >* 0.05; correlation heatmap, Figure 1H).

#### BAFF and APRIL levels are associated with inhibitor presence in adult HA patients.

**Figure 2:**
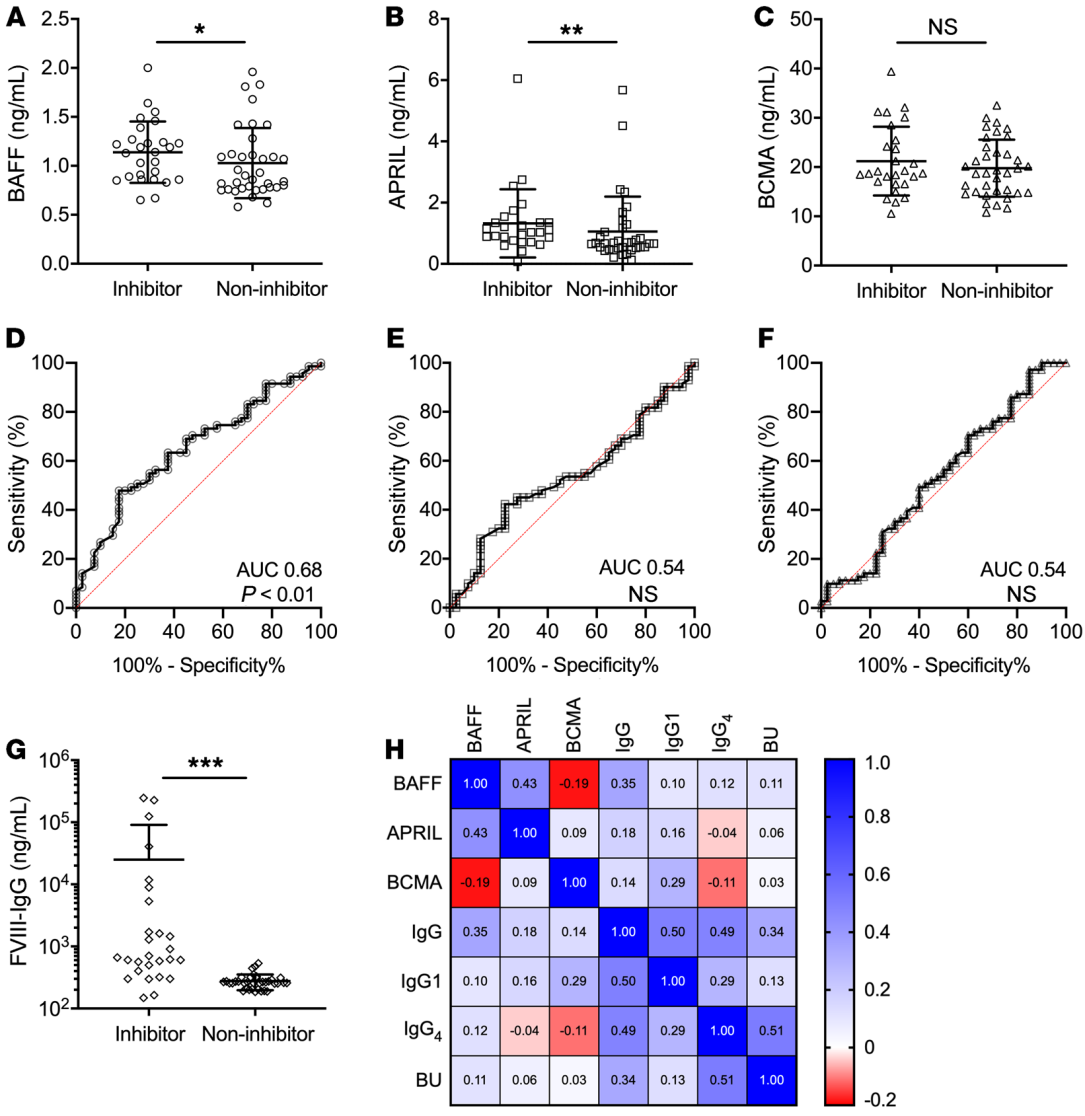
B cell cytokine levels in adult Italian hemophilia A patients. B cell cytokine and α-FVIII IgG levels in adult HA patients with (*n* = 22) or without (*n* = 24) FVIII inhibitors. (**A**) BAFF levels. (**B**) APRIL levels. (**C**) BCMA levels. Receiver operating characteristics of (**D**) BAFF, (**E**) APRIL, and (**F**) BCMA for pediatric and adult HA patients. (**G**) α-FVIII IgG in adult HA patients. (**H**) Spearman’s correlation heatmap of B cell cytokines and α-FVIII IgG in adult HA patients. *P < 0.05, **P < 0.01, ***P < 0.001 by Mann-Whitney *U* test. NS, not significant.

Recent genomic studies have identified 2 variants that lead to elevated BAFF levels and confer increased risk of autoimmune disease in patients of Italian, particularly Sardinian, descent (46). Thus, we sought to determine if HA inhibitor patients from Italy had increased B cell cytokine levels. Demographic data from 46 predominantly adult patients followed at the Careggi HTC are summarized in Table 1. Of the 46 patients, 22 (47.8%) had inhibitors and 24 (52.2%) did not have inhibitors. Of the 22 patients with inhibitors, 5 had achieved FVIII tolerance, 7 were on ITI, and 10 had failed ITI. Patient age did not differ between those with inhibitors (median 54, IQR 18–63 years) versus noninhibitor controls (median 44, IQR 28–57 years; *P* > 0.05 by Mann-Whitney *U* test). All patients were of Caucasian descent and there was no difference in disease severity or *F8* mutations between the cohorts. Levels of BAFF (1.14 ± 0.31 vs. 1.03 ± 0.36 ng/mL, *P* = 0.041) and APRIL (1.33 ± 1.11 vs. 1.06 ± 1.14 ng/mL, *P* = 0.008) were higher in patients with inhibitors versus noninhibitors (Figure 2, A and B). Levels of BCMA were not different between patients with and without inhibitors (Figure 2C). As expected, FVIII-specific IgG was considerably higher in the inhibitor cohort (25,039 ± 65,555 vs. 275.5 ± 77.8 ng/mL, *P* < 0.001; Figure 2G) and BAFF levels correlated with total IgG by Spearman’s correlation analysis (*ρ* = 0.35, *P* < 0.01; Figure 2H). The BAFF levels observed in the adult Italian HA inhibitor cohort were similar to those seen in the pediatric HA inhibitor cohort from the United States (1.14 ± 0.31 vs. 1.30 ± 0.61 ng/mL, respectively; *P* > 0.05) and higher than those in the noninhibitor pediatric cohort (1.14 ± 0.31 vs. 0.99 ± 0.47 ng/mL, respectively; *P* < 0.05). Although APRIL and BCMA levels trended higher in the pediatric inhibitor cohort, they were not statistically different between the inhibitor-positive adult and pediatric cohorts (*P* > 0.05). Noninhibitor adult Italian patients had lower APRIL levels (1.06 ± 1.14 vs. 2.97 ± 7.49 ng/mL, *P* < 0.05) and higher BCMA levels (20.66 ± 5.42 vs. 17.99 ± 4.08 ng/mL, *P* < 0.05) than the pediatric noninhibitor cohort.

Finally, we combined the pediatric and adult HA patient cohorts to determine whether BAFF, APRIL, and BCMA levels can be used to discern the presence of FVIII inhibitors by receiver-operating characteristic (ROC) analysis. ROC curves measure the probability of a test to distinguish a binary outcome at various thresholds and the area under the curve (AUC) represents the degree of separation. Thus, the higher the AUC, the more likely the test performs well in discerning disease state. In our analysis, the AUC was statistically significant for BAFF at 0.68 (95% CI 0.57–0.78, *P* < 0.01; Figure 2D) but not APRIL (AUC 0.54, Figure 2E) or BCMA (AUC 0.54, Figure 2F). Total operating characteristic curves of these cytokines are similar to the ROC curves (Supplemental Figure 2). BAFF levels greater than 1.03 ng/mL had 68.3% sensitivity, 63.8% specificity, and likelihood ratio of 1.89 for the presence of FVIII inhibitors, suggesting that BAFF could be a potential harbinger of an ongoing α-FVIII humoral immune response.

#### Increase in BAFF levels following rituximab therapy in adult and pediatric HA patients.

**Figure 3:**
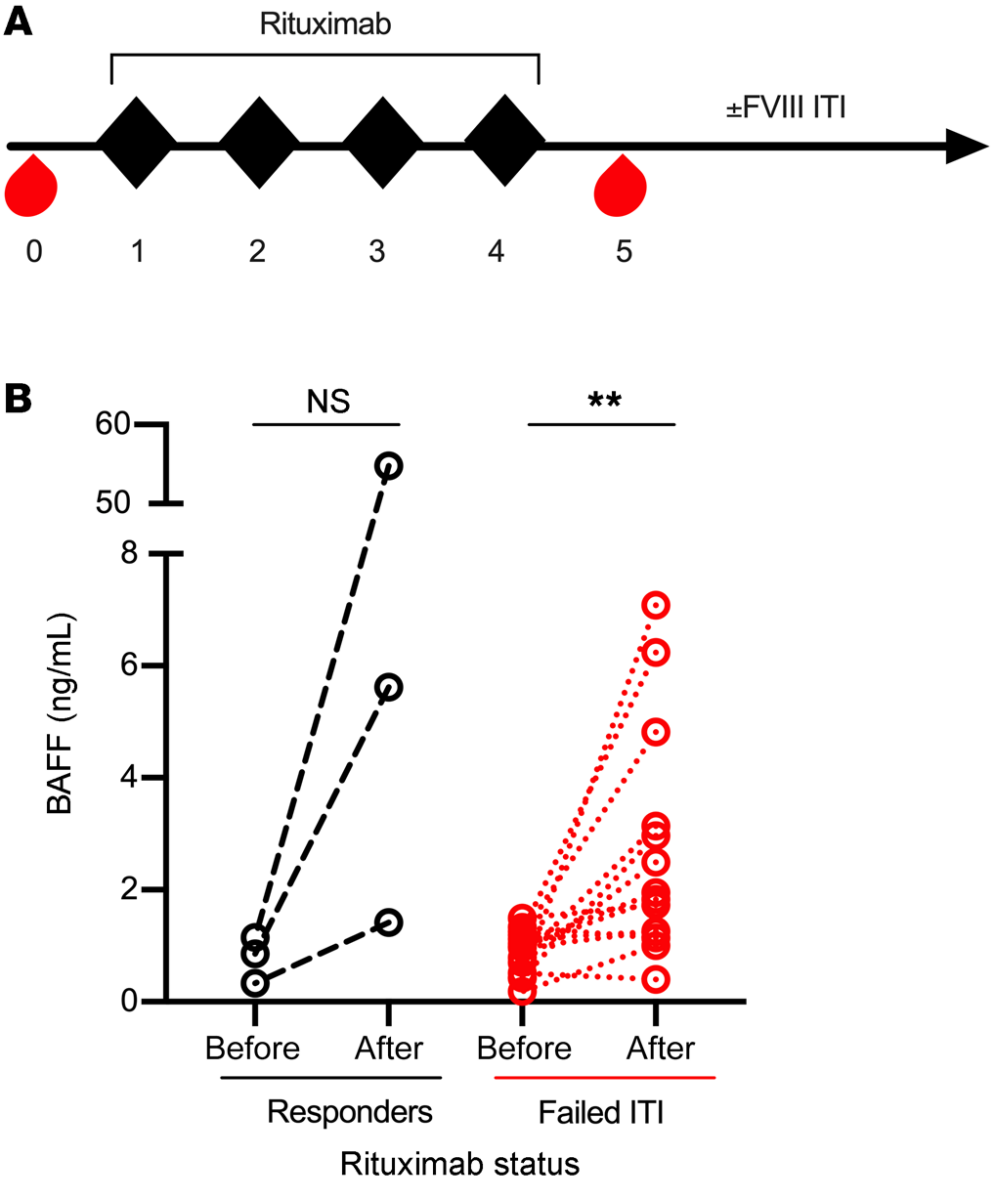
BAFF levels in HA inhibitor patients treated with rituximab. (**A**) Schema for rituximab therapy. Adult and pediatric HA patients with refractory inhibitors were treated with rituximab (black diamonds) and plasma samples (red drops) were obtained before and after therapy. Patients (*n* = 8) received concurrent FVIII ITI or not (*n* = 9). (**B**) BAFF levels before and after rituximab therapy in HA patients who did (black circles, *n =* 3) or did not (red circles, *n* = 14) achieve FVIII tolerance at the end of their ITI course. **P < 0.01 by paired *t* test. NS, not significant.

Next, we investigated whether a rise in BAFF after rituximab-based therapy precludes tolerance to FVIII, as seen in other allo- and autoimmune disease contexts. BAFF levels were measured from samples obtained from a total of 17 HA inhibitor patients. Of these, 9 were enrolled in the only prospective trial of rituximab alone for ITI-refractory FVIII inhibitors (RICH trial) wherein rituximab was dosed at 375 mg/m^2^ weekly for 4 weeks. The remaining patients were enrolled from HTCs at Emory University (*n =* 6) and CHOP (*n =* 2) who received the same dose of rituximab with concurrent FVIII protein replacement ITI. Plasma samples were obtained at baseline and following the last dose of rituximab (Figure 3A) and all patients were followed longitudinally for inhibitor titers. Of this cohort of 17 HA patients, 3 of 17 (17%) achieved tolerance to FVIII (1 treated with rituximab alone and 2 with rituximab and FVIII ITI), as defined by a negative Bethesda titer, and 14 of 17 (82%) did not achieve FVIII tolerance. Within the nonresponding cohort, 8 were treated with rituximab only and 6 were treated with rituximab and FVIII ITI. In the patients who failed to achieve FVIII tolerance (Figure 3B), BAFF levels increased 3-fold from baseline (0.89 ± 0.39 to 2.66 ± 2.03 ng/mL, paired *t* test *P* = 0.007). The relatively low number of patients who achieved FVIII tolerance prevented statistical conclusions, although levels did rise in this population as well (0.78 ± 0.42 to 20.60 ± 29.66 ng/mL, *P* > 0.05). In studies of other immune-mediated processes, the rise in BAFF following rituximab therapy has been shown to preclude antigen tolerance (54–56). Given the rise in BAFF in HA inhibitor patients treated with rituximab, we investigated this hypothesis in HA animal models.

### Inhibitor prevention and eradication studies in HA mouse models

We hypothesized that the elevated levels of BAFF in human HA inhibitor patients could serve as a survival signal for FVIII-reactive B cells and targeting BAFF may be of therapeutic value in FVIII inhibitors. Here, we tested the hypothesis that blocking BAFF could be effective in the prevention and/or eradication of FVIII inhibitors in animal HA models. Doering et al. have shown that the use of murine FVIII protein does not induce inhibitor formation in HA mice (57); to overcome this limitation, recombinant human FVIII (rhFVIII) protein concentrates are used. To avoid strain-specific results and limit the potential bias in the assessment of the immune responses, we used distinct HA strains on a C57BL/6-129 background (colony at CHOP) or BALB/c background (colony at Indiana University). The HA phenotype is similar between these strains but immune responses to pathogens, proteins, and gene therapy are known to differ (58–63). Further, we used 2 distinct murine α-BAFF (α-mBAFF) mAbs: (a) clone 10F4, which is a hamster IgG_1_ mAb with a half-life of approximately 2 weeks (64); and (b) clone Sandy-2, which is a mouse IgG_1_ mAb with a half-life of approximately 10 days (65). These antibodies are biologically equivalent in their inhibition of TR, follicular, and MZ B cells, with 10F4 taking longer (~8 weeks) for immune reconstitution compared with Sandy-2 (6 weeks) (64, 65).

#### α-mBAFF therapy prevents FVIII inhibitor development in FVIII-naive HA C57BL/6-129 mice.

**Figure 4:**
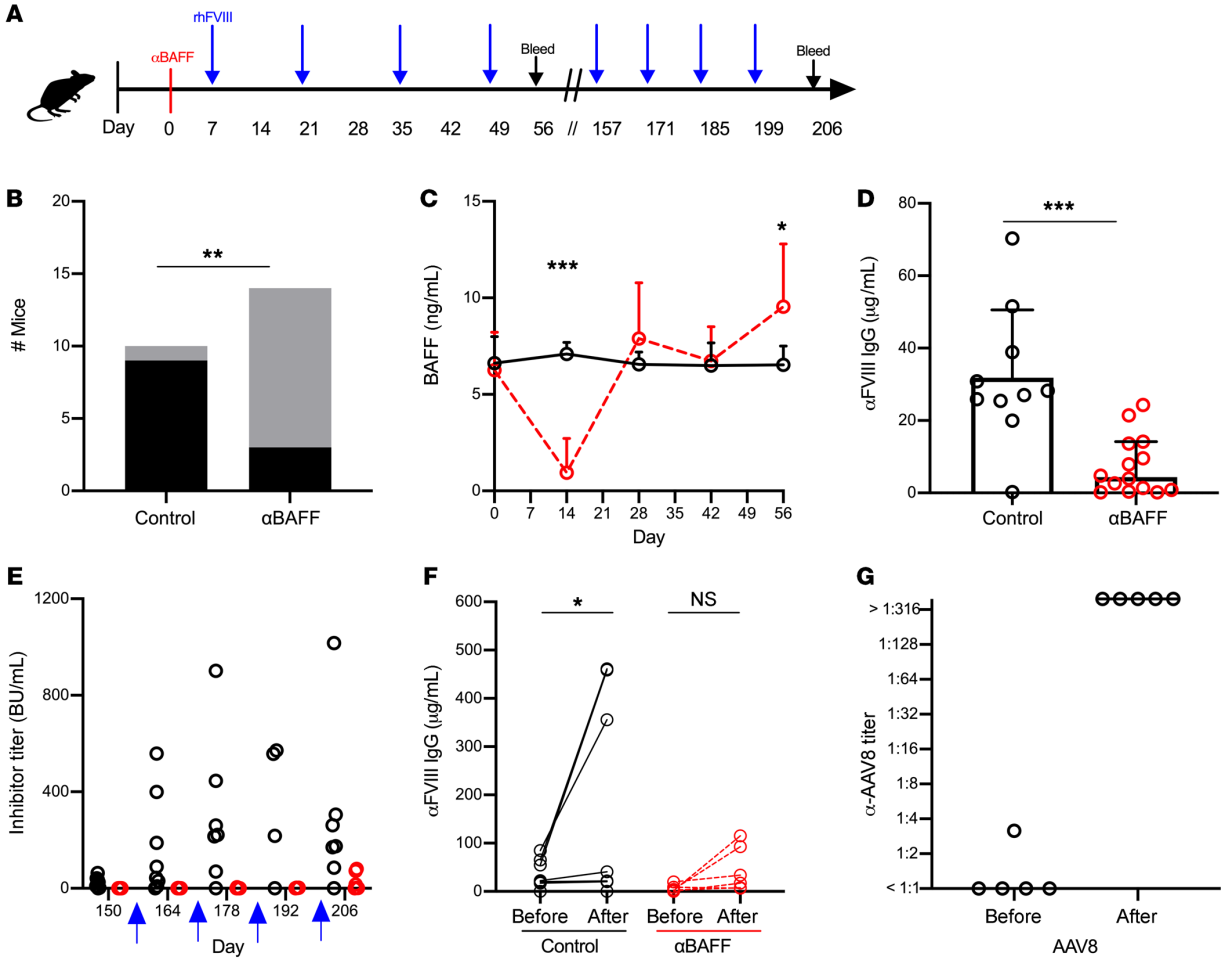
α-mBAFF antibody therapy for prevention of FVIII inhibitors in HA mice. (**A**) C57BL/6-129 HA mice (*n* = 10–14/group) were injected with α-mBAFF antibody prior to immunization with FVIII and followed longitudinally. (**B**) Number of inhibitor-positive (black bars) or -negative (gray bars) mice in controls versus α-mBAFF–treated groups. (**C**) BAFF levels over time in the α-mBAFF (red circles) and control (black circles) groups. (**D**) α-FVIII IgG in the α-mBAFF group (red circles) compared to controls (black circles) on day 56. FVIII inhibitor titers (**E**) and α-FVIII IgG (**F**) after remote FVIII challenge (blue arrow) in control (black circles) and α-mBAFF–treated (red circles) mice. (**G**) Titers of neutralizing antibodies against AAV8. AAV8 was injected 17 weeks after mice were treated with α-mBAFF antibody and α-AAV8 antibody titers were measured before and 4 weeks after AAV8 injection (*n* = 6). *P < 0.05; **P < 0.01; ***P < 0.001 by Fisher’s exact (**B**), 2-way ANOVA (**C**), Mann-Whitney *U* (**D** and **E**), Wilcoxon’s matched signed rank (**F**), and paired *t* (**G**) tests. NS, not significant.

As BAFF is necessary for the survival of TR and MZ B cells, the latter of which have been implicated in the initiation of the FVIII immune response in mice (30, 31), we investigated whether prophylactic α-mBAFF mAb therapy could prevent FVIII inhibitor formation in HA C57BL/6-129 mice, which mount a robust immune response to rhFVIII protein compared with BALB/c mice (63). FVIII-naive C57BL/6-129 HA mice (*n* = 10–14/group) were given α-mBAFF mAb (Sandy-2) or isotype control prior to immunization with rhFVIII and followed for FVIII inhibitor development (Figure 4A). Only 3 of 14 mice in the α-mBAFF group developed inhibitors, with Bethesda titers ranging from 0–150 BU with a median titer of 0 BU (IQR 0–0.5) compared with 9 of 10 mice in the control group with a range of 0–254 BU and median titer of 21.1 BU (IQR 2.5–157.3), resulting in a significantly reduced relative risk of 0.23 (95% CI 0.08–0.57) with α-mBAFF therapy (Figure 4B).

BAFF levels in α-mBAFF–treated mice were depleted at 14 days after injection (0.94 ± 1.78 vs. 7.10 ± 0.60 ng/mL, *P* < 0.001) and levels equalized by day 28 between groups (Figure 4C). α-FVIII IgG was lower in the α-mBAFF–treated group (7.52 ± 8.07 μg/mL) compared with controls (31.83 ± 18.77 μg/mL, *P* < 0.001; Figure 4D). Of note, these experiments were also conducted in HA BALB/c mice, a model that requires weekly rhFVIII immunization to mount an inhibitor response. The data showed decreased α-FVIII IgG, with a median of 0.19 (IQR 0.12–0.52) in treated versus 0.75 (IQR 0.42–2.43) μg/mL in control mice (*P* < 0.05) (data not shown).

In the HA C57BL/6-129 mice, 22 weeks after initial α-mBAFF mAb, long-term tolerance to FVIII was tested by rhFVIII injections in 6 mice from the α-mBAFF treatment group (Figure 4E). No mice developed a high-titer (BU > 5) FVIII inhibitor response after the first challenge and, thus, immunizations were continued for a total of 4 challenges. After the fourth challenge, control mice had a median inhibitor titer of 174.6 BU (IQR 85.6–305.6) compared with 8.5 BU (IQR 0.9–74.1) in α-mBAFF–treated mice (*P* < 0.05). Remarkably, only half of the mice from the α-mBAFF group developed high-titer inhibitors (15–80 BU), whereas the remaining mice had inhibitor titers of less than 2 BU. Corresponding FVIII-specific IgG levels before and after the 4 challenge rhFVIII doses are presented in Figure 4F. Thus, a single dose of α-mBAFF was sufficient to prevent the formation of high-titer inhibitors in HA mice, with a sustained effect (>22 weeks) beyond the relative short initial period of reduction of BAFF levels (4 weeks). To ensure that mice were capable of mounting an immune response to a T cell–dependent antigen, a second cohort of α-mBAFF–treated mice were challenged with adeno-associated virus type 8 (AAV-8) vector at 17 weeks and developed robust neutralizing antibody responses, with titers higher than 1:316 dilution (Figure 4G). Thus, the lack of a robust neutralizing antibody responses with FVIII challenge in these mice suggests that prophylactic α-mBAFF mAb therapy during initial FVIII exposure may bias the immune system specifically toward FVIII-antigen tolerance.

#### Combination α-mCD20/α-mBAFF mAb therapy induces tolerance in HA BALB/c mice with established FVIII inhibitors.

**Figure 5:**
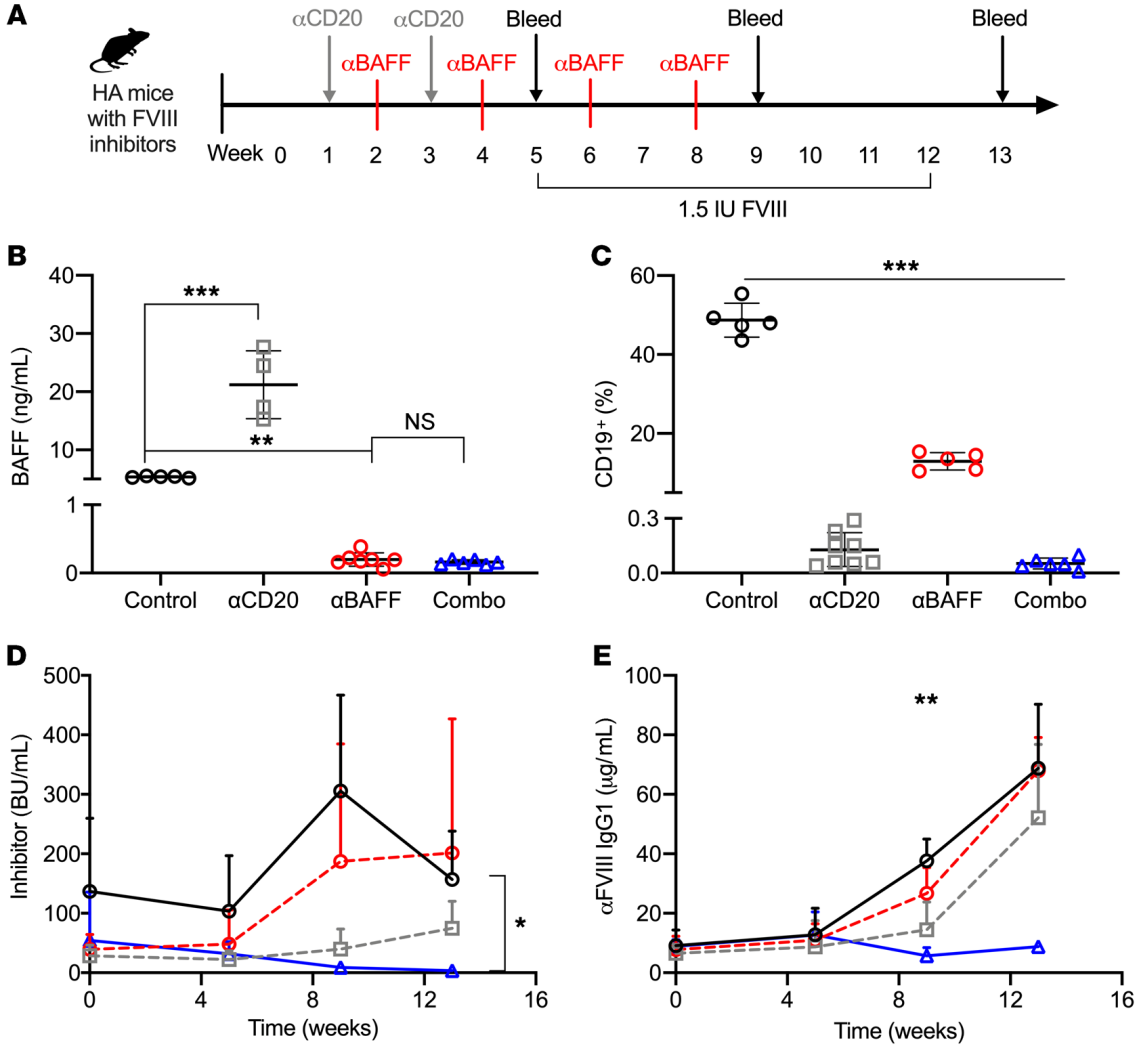
Combination of α-mBAFF and α-mCD20 therapy for FVIII tolerance induction. (**A**) Schema for combination α-mCD20 and α-mBAFF therapy. HA-BALB/c mice with established inhibitors were treated with α-mCD20 (gray squares, *n* = 8), α-mBAFF (red circles, *n* = 6), combination therapy (blue triangles, *n =* 6), or no treatment (black circles, *n* = 5) and followed for 13 weeks. (**B**) BAFF levels at week 5, (**C**) peripheral CD19^+^ B cells at week 5, (**D**) inhibitor titer, and (**E**) α-FVIII IgG_1_. *P < 0.05; **P < 0.01; ***P < 0.001 by 1-way ANOVA (**B** and **C**) or mixed-effects ANOVA (**D** and **E**). NS, not significant.

The clinical burden of disease in HA resides with patients with established inhibitors. Thus, we sought to determine if α-mBAFF–based therapy could be effective in eradicating FVIII inhibitors. HA BALB/c mice with inhibitors were treated with α-mCD20 alone, α-mBAFF alone (GlaxoSmithKline clone 10F4), or combination α-mCD20/α-mBAFF mAb therapies, as depicted in Figure 5A. Recapturing the human data following rituximab therapy, HA-BALB/c mice treated with an α-mCD20 antibody had a 3-fold increase in mBAFF levels at week 5 (Figure 5B) compared with control mice (15.57 ± 0.47 vs. 5.37 ± 0.15 ng/mL, *P* < 0.001), and this rise was ameliorated by addition of α-mBAFF mAb to α-mCD20 (0.16 ± 0.34 ng/mL, *P* < 0.001 vs. α-mCD20 alone), which was similar to α-mBAFF mAb alone. APRIL levels were not elevated (data not shown). Compared with control mice, all treatment group mice had lower peripheral CD19^+^ B cell percentages at week 5, with levels of 48.7 ± 4.3 in controls, 0.1 ± 0.1 in α-mCD20, 12.9 ± 2.2 in α-mBAFF, and 0.1 ± 0.0 in combination therapy (*P* < 0.001 by 1-way ANOVA, Figure 5C). Only the combination therapy resulted in a substantial decrease in inhibitor titer (3.7 ± 1.7 vs. 156.9 ± 81.3 BU, *P* < 0.05) and α-FVIII IgG_1_ (8.28 ± 1.46 vs. 68.76 ± 21.51 μg/mL, *P* < 0.01) compared with controls (Figure 5, D and E), even following repeated rhFVIII challenge. From prior experiments, B cell repopulation was seen by 4 weeks after α-mCD20 mAb administration, so these challenges occurred during or after B cell recovery (66). Thus, the combination therapy of α-mBAFF mAb and α-mCD20 is effective in eradicating FVIII inhibitors and maintaining immune tolerance despite continued challenges with the protein. In the more immunogenic HA C57BL/6-129 mice, inhibitor titers decreased from 70 BU to less than 5 BU with 2 cycles of this combination therapy regimen (data not shown).

For preliminary quantification of immune cell subsets involved in the induction of tolerance to FVIII, HA BALB/c mice were treated with the various mAb regimens to monitor B cell repopulation at weeks 5 and 9 (Supplemental Figure 3, A–F). Compared with control mice, spleens of combination-treated mice had universally lower B cell subsets, including follicular, MZ, MEM, TR, and PCs as well as plasmablasts at week 5 (*P* < 0.001), of which follicular (*P* < 0.001), MZ (*P* < 0.01), and PC (*P* < 0.05) depletion persisted at week 9. Consistent with previous reports (67–69), BAFF-R expression was present in follicular and MZ B cell subsets, splenic plasmablasts, and PCs, with highest expression in GC B cells and lower expression in BM plasmablasts and PCs (Supplemental Figure 3G). In contrast, TACI expression was highest in splenic and BM plasmablasts and PCs (Supplemental Figure 3H). These initial data suggested that a PC-dependent mechanism was responsible for the ability of combination therapy to induce FVIII tolerance. Either BAFF or APRIL can support PC survival in the BM, indicating a redundant role for BAFF in LLPC maintenance (70).

#### α-mBAFF performs similarly to mTACI-Fc in combination with α-mCD20 for FVIII inhibitor eradication.

**Figure 6:**
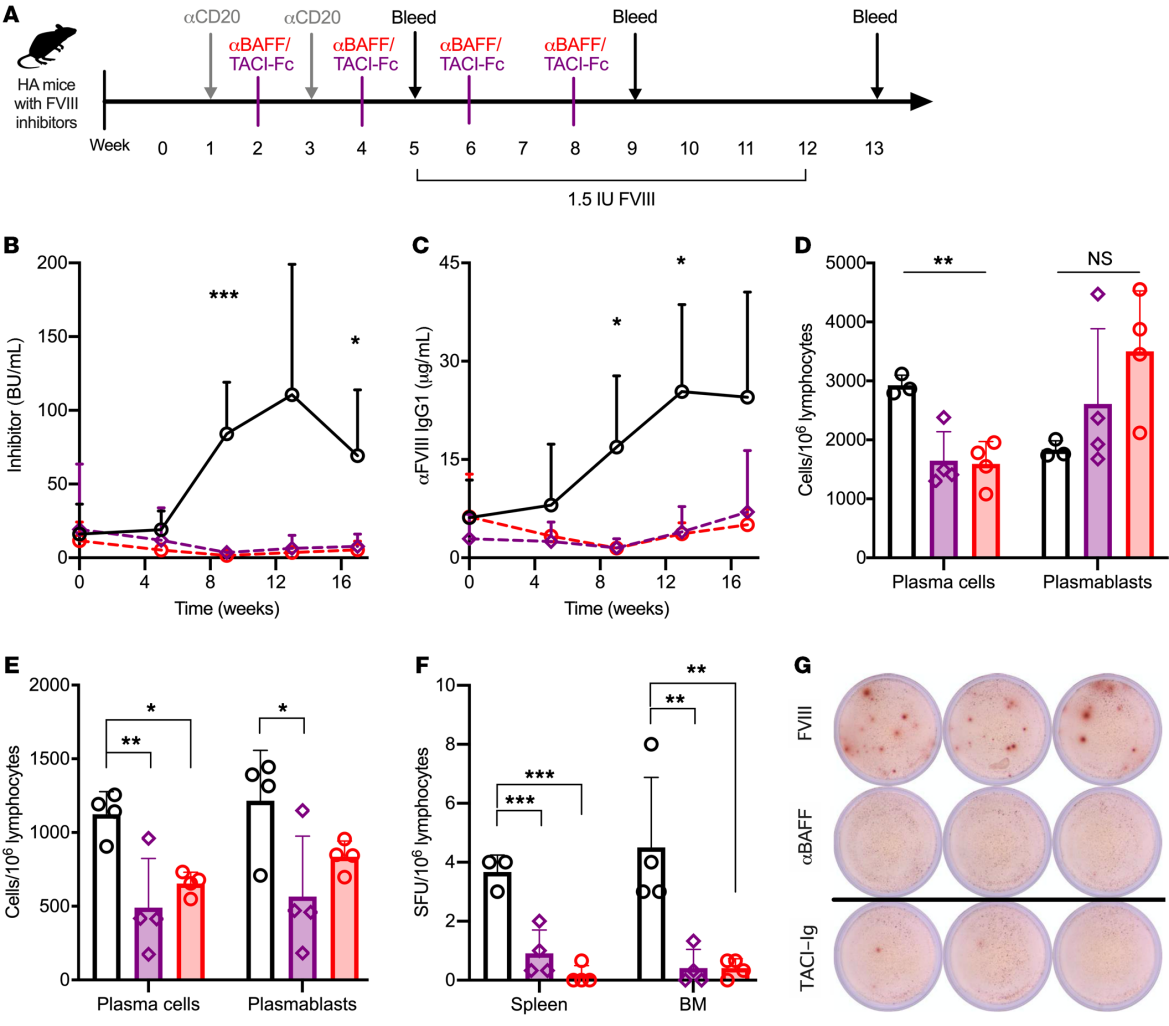
Combination therapy with α-mCD20 with α-mBAFF or mTACI-Fc in FVIII inhibitor mice. (**A**) Schema for combination α-mCD20 and α-mBAFF or mTACI-Fc therapy. HA inhibitor mice were treated with α-mCD20 with α-mBAFF (red circles, *n* = 10), α-mCD20 with mTACI-Fc (purple diamonds, *n* = 8), or no treatment (black circles, *n =* 8) and followed for (**B**) Bethesda titer and (**C**) α-FVIII IgG_1_. At 16 weeks from start of regimen, spleens (**D**) and bone marrow (**E**) were harvested for quantification of plasmablasts and plasma cells by flow cytometry (values normalized per million lymphocytes). (**F**) FVIII-specific B cell ELISPOT from splenic and bone marrow plasma cells (conducted in triplicate from *n* = 4 mice per group), with representative images of samples (**G**). *P < 0.05; **P < 0.01; ***P < 0.001 by mixed-effects ANOVA (**B** and **C**) or 1-way ANOVA (**D**–**F**). NS, not significant.

To investigate the PC compartment further, we compared performance of α-mCD20 mAb with α-mBAFF or mTACI-Fc mAb. Consisting of the Fc region of IgG and the binding domain of the TACI receptor, mTACI-Fc (atacicept in humans, ref. 71) can bind and inactivate both BAFF and APRIL in their soluble forms and thereby inhibit downstream signaling (72). As TACI-Fc is known to target PC survival (70), similar results between these 2 mAb therapies would confirm that combination therapy exerts its effect through a PC-mediated process. Littermate controlled HA BALB/c mice with preexisting inhibitors were treated with α-mCD20 and α-mBAFF or mTACI-Fc mAb, as delineated in Figure 6A. Compared with control mice, α-mCD20 plus mTACI-Fc or α-mBAFF mAb resulted in significantly lower inhibitor titers starting at 2 months (84.1 ± 34.9, 3.5 ± 3.5, and 1.6 ± 1.8 BU, respectively; *P* < 0.001) but no difference was seen between the mBAFF- and mTACI-targeted groups (Figure 6B). A higher proportion of α-mBAFF mice had low-titer inhibitors (BU < 5) at week 13 compared with mTACI-Fc (88.9% vs. 62.5%, respectively) but this was not statistically significant. Similarly, the α-FVIII IgG_1_ titer (Figure 6C) was higher in control mice compared with the α-mBAFF or mTACI-Fc mice at 2 months (16.9 ± 10.9 vs. 1.5 ± 1.4 or 1.5 ± 0.7 μg/mL, respectively; *P* < 0.001). Tolerance was sustained for the duration of the experiment (4 months), even after weekly immunological challenges with rhFVIII protein (months 2 and 3). These data support our findings that combination therapy targeting mCD20 and mBAFF eradicates FVIII inhibitors through a PC-mediated process.

#### Combination therapy targeting mBAFF or mTACI suppresses PCs.

To determine the effect of combination therapies on PCs, spleens and BM were harvested from mice at the 4-month time point for flow cytometric quantification of PCs and plasmablasts and an FVIII-specific B cell ELISPOT assay. Mice treated with α-mBAFF or mTACI-Fc had a reduction in splenic PCs compared with controls (1593 ± 379, 1646 ± 49, and 2927 ± 171 cells/10^6^ lymphocytes, respectively; *P* < 0.01) but not splenic plasmablasts (3501 ± 1026, 2611 ± 1273, and 1836 ± 150 cells/10^6^ lymphocytes, respectively), as seen in Figure 6D. There was a decrease in BM PCs (653 ± 77, 491 ± 332, and 1124 ± 152 cells/10^6^ lymphocytes, respectively, *P* < 0.01; Figure 6E) across all groups. BM plasmablast counts were higher in control (1216 ± 341) versus mTACI-Fc–treated mice (565 ± 411, *P* < 0.05) but not α-mBAFF mice (837 ± 107 cells/10^6^ lymphocytes). These results were consistent with ELISPOT analysis, which showed fewer spot-forming units (SFU) in the experimental versus treated mice (Figure 6, F and G). Combined with data from earlier time points (Supplemental Figure 3), these data suggest that combination mAb treatment could bias the immune system toward FVIII immune tolerance via sustained depletion of FVIII-specific PCs.

## Discussion

FVIII is one of the most immunogenic biologics (1) and inhibitors of FVIII pose a significant barrier to optimal care, thereby increasing patient morbidity and mortality. Understanding mechanisms that predispose and/or drive FVIII immune responses is thus of clinical significance. ITI protocols are not always effective at eradicating inhibitors and combination immunosuppressive strategies to date have had meager results, with toxicity-related limitations. The α-CD20 mAb rituximab held promise for FVIII inhibitor eradication in early reports, but the modest efficacy observed in a prospective study of monotherapy with rituximab (26) has dampened this optimism. This suboptimal efficacy may partially be explained by the persistence of rituximab-resistant FVIII^+^ B cell subsets. In the context of autoimmune cytopenias, rituximab paradoxically promotes the rapid repopulation of cells with an LLPC phenotype in extrafollicular foci in the spleen (54, 73, 74). Further, increased availability of BAFF as a result of B cell depletion is thought to possibly exacerbate autoimmune disease in patients (55, 56) and increase alloantibody responses in graft-versus-host disease (75) or transplant rejection (51). Thus, we hypothesized that BAFF plays a role in the initiation and/or maintenance of the FVIII immune response, and so we used distinct HA patient samples and mouse models to address this hypothesis. Our data show a potential role for BAFF inhibition in both prevention and eradication of FVIII inhibitors.

Using a rare cohort of longitudinal samples from pediatric HA patients, we show that levels of BAFF are elevated in pediatric HA inhibitor patients and a decrease in BAFF correlates with successful FVIII tolerance induction with ITI. The α-FVIII IgG response in humans is characterized by both low- and high-affinity antibodies, which correspond to nonneutralizing and neutralizing antibodies, respectively (76, 77). Our pediatric HA patient data indicate that BAFF levels correlate most strongly with the FVIII-inhibitor and neutralizing-IgG_4_ titers but also with α-FVIII IgG_1_, whereas other T-helper cytokines tested here did not. Prior studies have shown correlation of polymorphisms in regulatory elements of certain cytokines with inhibitors but few have assessed cytokine levels; the results from either method are inconsistent between populations of distinct geographic origins (78–82). As genetic polymorphisms that predispose patients to elevated BAFF levels have been characterized in people of Sardinian and continental Italian descent compared with northern Europeans (46), we measured BAFF levels from an Italian adult HA cohort. BAFF and APRIL levels were elevated in our cohort of adult Italian HA inhibitor patients. Comparing the Italian adult to the US pediatric inhibitor-positive cohort, levels of BAFF, APRIL, and BCMA did not differ between the 2 sites. However, in noninhibitor patients, APRIL levels were higher and BCMA levels were lower in the pediatric US cohort compared with the Italian adult cohort. Of note, APRIL and BAFF levels are known to decrease with age (83) and are thought to help maintain the peripheral B cell pool, which also decreases with age (84). The similarity of the BAFF levels between adult and pediatric inhibitor patients, thus, suggests that ongoing elevated BAFF may contribute to circulating α-FVIII B cells. Naive B cells and plasmablasts rely on APRIL for survival, which may explain the higher APRIL levels in pediatric patients (85). Nevertheless, upon combination of these 2 groups (*n* = 115), BAFF had an AUC of 0.68 in the ROC analysis for inhibitor presence and greater than 60% sensitivity and specificity for inhibitors at a cutoff of 1.03 ng/mL. Although statistically significant, the differences in the adult and pediatric populations do need to be taken into account in using BAFF levels as a diagnostic marker of inhibitor presence. Rather, ongoing elevation or a rise in BAFF levels over time in a patient with an inhibitor could potentially serve as a marker of incomplete tolerance induction (given its correlation with α-FVIII IgG) or harbinger of impending ITI failure.

The data suggest a role for BAFF as a modulator of FVIII inhibitors but the underlying mechanism and timing of BAFF elevation remains to be determined. Presentation of FVIII during times of immune activation is thought to increase the likelihood of inhibitor development (86). Correlation of BAFF with the proinflammatory cytokines IFN-γ and IL-2 in the pediatric cohort may support the idea that BAFF participates in this cascade to elicit a strong immune response to FVIII. An alternative hypothesis is that BAFF levels are elevated prior to inhibitor development and serve as an adjuvant to FVIII. Certainly, coadministration of BAFF protein with vaccines has been shown to increase antibody titers (87). Finally, BAFF levels could be a surrogate marker of unregulated B cell activity. The continued high levels of BAFF seen in adult inhibitor patients and in pediatric patients who fail ITI would support this possibility.

Additionally, utilizing rare plasma specimens from refractory HA inhibitor patients who received rituximab-based ITI, we show that those who fail to establish tolerance to FVIII have BAFF levels that rise 3-fold from baseline, a finding that is mirrored in HA mice that receive α-mCD20 mAb therapy. Together, our data suggest that BAFF may be associated with the α-FVIII B cell response. Further, as seen in the 6 successfully tolerized patients in the pediatric cohort, BAFF levels in conjunction with α-FVIII IgG could potentially be used as a surrogate for likelihood of successful ITI. Identification of BAFF modifiers could provide additional insight into the reason behind this elevation.

Next, we establish that a single dose of α-mBAFF mAb in naive HA mice prevents inhibitor development (even after immune reconstitution) and that, in mice with preexisting inhibitors, the combination of α-mBAFF and α-mCD20 dramatically reduces and/or eliminates FVIII inhibitors, with sustained suppression of FVIII-specific PCs. Recent evidence suggests that the initial FVIII immune response is mediated by MZ B cells (30, 31), which can rapidly differentiate into short-lived antibody-secreting cells (ASCs) (88). However, high-affinity antibodies typically result from GC reactions that result in BCR rearrangement, leading to differentiation into PCs or MEM B cells. Within GCs, Tfh cells secrete BAFF in order to promote the selection of high-affinity GC B cell clones (69, 89). In HA inhibitor mice, Reipert’s group has shown that MEM B cells can drive ASC generation (90). Although rituximab is thought to work primarily by suppressing MEM B cells, the poor response to rituximab ITI in HA patients suggests that MEM B cells are not solely responsible for the FVIII immune response. This finding is supported by additional murine studies in which depletion of PCs was necessary for long-term tolerance induction (91). Additional studies are needed to further confirm these findings.

We hypothesize that combination therapy with α-CD20 and α-BAFF prevents the selection of high-affinity B cell clones in the GC and depletes FVIII-specific PCs. In our study, neutralization of downstream signaling from BAFF and APRIL via combination therapy with α-mCD20 and mTACI-Fc did not additionally improve inhibitor eradication over combination with α-mBAFF. As TACI-Fc is known to target PC survival and spare MEM B cells, consistent with the PC depletion seen in our experiments, this supports the role of PCs in the FVIII immune response. Although inhibitor titers were still dramatically low in the combination therapy groups at 16 weeks, there was a small increase in the titer between weeks 12 and 16 (when further FVIII was not given). This may point to a resurgence of MEM B cells that are driving new PC generation, as suggested by the data from Reipert et al. (90). Notably, however, low BU titers (≤5) were sustained at the 16-week time point, which would allow inhibitor patients to resume FVIII replacement therapy, which is the main goal of successful ITI.

In a trial of kidney transplant recipients who received the α-BAFF mAb belimumab in an effort to decrease de novo IgG production and limit allograft rejection, MEM B cell numbers were not different between the belimumab-treated and control groups (92). However, de novo IgG production still dropped 3-fold in the belimumab group even after discontinuation of therapy and was associated with a skewing of cytokine production favoring IL-10 over IL-6 in TR and MEM B cells, thus supporting a tolerogenic immune profile. Finally, the lack of tolerance induction in preexisting-inhibitor mice with α-BAFF therapy alone mimics the human experience in HLA sensitization (93). BAFF is not known to affect preexisting MEM B cells in isolation without α-CD20 therapy. Thus, the mechanism of success with α-CD20 and α-BAFF combination therapy is likely the combination of initial MEM B cell depletion with α-CD20 therapy followed by prevention of new FVIII^+^ PCs by α-BAFF therapy.

The dramatic reduction in inhibitor levels with preemptive α-mBAFF mAb is explained by the reliance of MZ B cells on BAFF for survival and differentiation (94). We postulate that these mice have lower rates of GC B cell reactions and consequently fewer PCs and MEM B cells, allowing for tolerance to FVIII. This is supported by the fact that nearly half of these mice did not generate a high-titer immune response despite 4 remote FVIII challenges after immune reconstitution, suggesting that FVIII exposure during B cell reconstitution after initial mAb therapy may have shifted the immune balance toward tolerance. Notably, these mice are able to mount a robust immune response against an unrelated antigen, supporting the safety and specificity of this strategy. Our data, combined with studies in enzyme replacement therapy (95), indicate that BAFF plays a role in the immunogenicity of biotherapeutics. As some HA mice still developed a high-titer α-FVIII antibody response, future studies are needed to determine whether modification of dose and/or treatment duration would help prevent inhibitors completely. Certainly, before translational studies, additional data regarding the safety of this regimen are necessary, especially as patients with inhibitors are typically less than 2 years of age. Using a specific immune modulatory strategy in these young patients may be safer than general immunosuppressive regimens tested in hemophilia (21, 24, 96) and other genetic diseases (97–100).

Our study does have some limitations. First, there are likely differences in the established, longer immune response seen in adults versus pediatric inhibitor patients whose immune responses may still be evolving, as noted in the HIPS study (34). However, the continued elevation of BAFF in the adult HA inhibitor population (which otherwise should fall) supports the hypothesis that BAFF modulates the FVIII immune response. Second, mice are genetically more homogeneous in comparison with humans; we attempted to ameliorate this by using a variety of antibody reagents and 2 different HA strains. Finally, although common to all small-animal HA models, the rhFVIII immune response studied in mice is a xenoprotein response and thus may not be directly applicable to the human experience. However, as both are alloantibodies and given that most inhibitor patients do not make endogenous FVIII, we think characterizing the response to rhFVIII in a mouse model provides valuable insight into both inhibitor formation and potential therapeutics targeting human α-FVIII antibodies.

In summary, our data establish the potential to use α-BAFF therapy in conjunction with α-CD20 therapy for eradication of FVIII inhibitors in patients with HA. Belimumab is FDA approved in pediatric and adult patients and trials of combination therapy with rituximab are ongoing in autoimmune disease contexts in adults (NCT02631538, NCT02260934, NCT03967925, NCT03747159, etc.). These data, along with pediatric trial data, could provide important safety information for use in young inhibitor patients. Future studies aimed at understanding the longevity and exact mechanism of this response are needed. Determining whether genetic variants in BAFF (46) or other BAFF modifiers are present in HA inhibitor patients could help identify those at high risk of inhibitor development and/or ITI failure. Finally, as HA patients enrolled here were treated both with recombinant and plasma-derived FVIII concentrates, BAFF levels seem to be associated with inhibitor development in general. Proving this concept with the highly immunogenic FVIII protein could allow for expansion of this strategy for other diseases complicated by an immune response to biotherapeutics: for instance, hemophilia B and/or enzyme replacement therapy in other genetic diseases.

## Methods

### HA patients.

Pediatric HA patients (*n =* 69) were recruited consecutively from CHOP and predominantly adult HA patients from Careggi University Hospital (*n =* 46) HTCs. In addition, adult and pediatric HA patients treated with rituximab for ITI-refractory inhibitors were recruited from the phase II trial “Rituximab for the Treatment of Inhibitors in Congenital Hemophilia A” (RICH trial, NCT00331006, *n =* 9; ref. 26), CHOP (*n =* 2), and Emory University (*n =* 6). Patients received 4 doses of 375 mg/m^2^ rituximab alone (RICH) or rituximab with FVIII ITI (CHOP and Emory). Each patient’s baseline pre-rituximab sample served as their internal control. Patients were enrolled in the RICH trial if they had severe congenital HA, were over 18 months of age, and had a historical Bethesda titer of greater than 5 BU and excluded if they had received immunomodulatory therapy within 30 days. Patients opted to enroll in a separate biorepository and only these patients who had samples from before and after rituximab therapy were included in the present study.

Specimens were processed within 1 hour of blood collection. Citrated plasma was aliquoted and frozen at –80°C until ready for analysis.

### Animal studies.

*F8* exon 16–knockout hemophilic mice were on a BALB/c background (BALB/c *F8e16^–/Y^*) bred at Indiana University (gift from David Lillicrap, Queen’s University, Kingston, Ontario, Canada) or on a C57BL/6-129 background bred at CHOP (gift from Haig Kazazian, University of Pennsylvania). Animal studies were done using littermate controls.

For inhibitor eradication experiments, 8- to 10-week-old HA BALB/c mice (*n =* 5–8/group) were immunized i.v. with 1.5 IU B domain–deleted rhFVIII (BDD-rhFVIII) (Pfizer) weekly to establish inhibitors (28–136 BU). Mice were subsequently treated with (a) 250 μg α-mCD20 mAb at 21-day intervals for 2 doses, (b) α-mBAFF mAb at 2.8 mg/kg at 14-day intervals for 2 doses followed by 1.6 mg/kg every 14 days for 2 doses, (c) α-mCD20 followed by α-mBAFF, or (d) no treatment (Figure 5A). α-mCD20 IgG_2a_ (clone 18B12) was purified from transfected HEK293 cells (ATUM) (101) and α-mBAFF mAb (clone 10F4) was from GlaxoSmithKline (64). All animals received weekly 1.5 IU BDD-rhFVIII i.v. from weeks 5 to 12. BM and spleens were harvested from *n =* 4 mice at weeks 5 and 9 for lymphocyte subset analysis. Data were analyzed by FlowJo version 10 (FlowJo LLC) or FCS Express 7 (De Novo Software). Flow cytometric antibody panels, lymphocyte subsets, and details can be found in Supplemental Table 1, with the gating strategy shown in Supplemental Figure 4. Each panel included fluorescence minus one, single-stained, and negative controls. In a parallel set of experiments, HA BALB/c mice with established inhibitors were treated with mTACI-Fc antibody (Biolegend) at 2.8 mg/kg every 2 weeks for 4 doses starting 1 week after α-mCD20 mAb. Animals were followed longitudinally for α-FVIII IgG_1_ and Bethesda titer and sacrificed at 4 months for quantification of lymphocyte subsets and enzyme-linked immunospot (ELISPOT) analysis.

For inhibitor prevention experiments, 8- to 12-week-old HA C57BL/6-129 mice (*n =* 10–14/group) were treated with either α-mBAFF mAb (65) or IgG_1_ isotype control (Adipogen) at 2 mg/kg once and subsequently immunized every 2 weeks with rhFVIII (Takeda Pharmaceuticals) i.v. at 2 IU for 4 injections. Four additional rhFVIII challenges were conducted at 22 to 30 weeks to test longevity of tolerance induction. Retro-orbital blood was collected longitudinally to monitor BAFF and α-FVIII antibody titers.

### FVIII antibody ELISA.

Murine IgG antibodies against FVIII were detected using an ELISA as previously described (66, 102, 103). Human α-FVIII IgG_1_ and IgG_4_ ELISAs were performed as described by Whelan et al. (77), with minor modifications as detailed in Supplemental Methods.

### Bethesda assay.

For human samples, Bethesda titers from citrated plasma specimens were quantified after heat inactivation at 56°C for 30 minutes to remove residual FVIII (104). Murine samples were used directly without heat inactivation. Bethesda assays were conducted as previously described (66, 103). Bethesda titer was calculated as percentage residual activity against a known noninhibitor control (5); levels greater than 0.6 BU were considered positive.

### Cytokine levels.

BAFF levels from mouse plasma samples were measured by ELISA (R&D Systems) per the manufacturer’s instructions (105). Peripheral IFN-γ, TNF-α, IL-2, IL-4, and IL-10 levels were measured by a customized Luminex bead array (MilliporeSigma) following the manufacturer’s instructions (106). BAFF, APRIL, and BCMA levels from the CHOP and Careggi patient samples were measured by a multiplex ELISA at the University of Pennsylvania Translational and Correlative Studies Laboratory (see Supplemental Methods for details). Because of limitations on sample transport, BAFF levels from rituximab-exposed patients were measured by ELISA (R&D Systems) per the manufacturer’s instructions (107) with controls to normalize data between laboratories.

### ELISPOT assays.

The frequency of FVIII-specific immunoglobulin-secreting B cells was quantified by a B cell ELISPOT assay as described previously (108). Briefly, RBCs from splenocyte or BM single-cell suspensions were lysed (eBioscience) and double filtered through 70-μm cell strainers. Cells (1 × 10^6^) were seeded in triplicate in RPMI 1640 plus 10% FBS onto B cell ELISPOT–specific plates (Millipore) precoated with BDD-rFVIII (2 g/mL). After overnight incubation at 37°C in 5% CO_2_, cells were removed by washing in PBS plus 0.5% Tween 20. Rat α-mIgG_1_–HRP (AbD Serotec) was used for detection followed by addition of AEC substrate (BD Biosciences) for spot development. Plates were analyzed using the ImmunoSpot system (Cellular Technology Limited).

### Statistics.

Data were analyzed using GraphPad Prism version 8. Patient demographic data were analyzed by χ^2^ analysis (for categorical variables) or Mann-Whitney *U* test (for continuous variables). Spearman’s correlation was used to analyze cytokine levels with FVIII inhibitor titer and α-FVIII IgG. Comparison of 2 groups was done by *t* tests (with paired *t* test for before/after intervention experiments) or Mann-Whitney *U* test. Parametric versus nonparametric tests were used after normality was tested using the Shapiro-Wilk test. For multiple group comparisons, 1-way ANOVA was used for single-time-point studies and repeated-measures mixed-effects ANOVA was used for longitudinal studies, both with Tukey’s correction for multiple comparisons. Relative risks were calculated by Fisher’s exact test. Data are presented as mean ± SD unless otherwise stated, with *P* less than 0.05 considered significant.

### Study approval.

Human subject investigation was done according to Declaration of Helsinki principles and was approved by the IRBs of CHOP (IRB08-7008) and Emory (IRB00006290). At Careggi University Hospital, written consent was obtained from patients to use stored samples for research purposes. Data use and material transfer agreements were approved by CHOP for use of the RICH trial samples. All animal studies were conducted in accordance with the GlaxoSmithKline policy on the care, welfare, and treatment of laboratory animals (https://www.gsk.com/media/2936/care-welfare-and-treatment-of-animals-policy.pdf) and were approved by respective IACUCs (CHOP IAC20-001269, Indiana University 18037).

## Author contributions

BSD, JR, MAS, RK, and JSSB conducted the experiments. BSD and MB designed the experiments and analyzed data. GC, SLM, and CL provided samples. BSD, MB, and VRA wrote the manuscript. MB and VRA directed the study.

## Supplementary Material

Supplemental data

## Figures and Tables

**Table 1:**
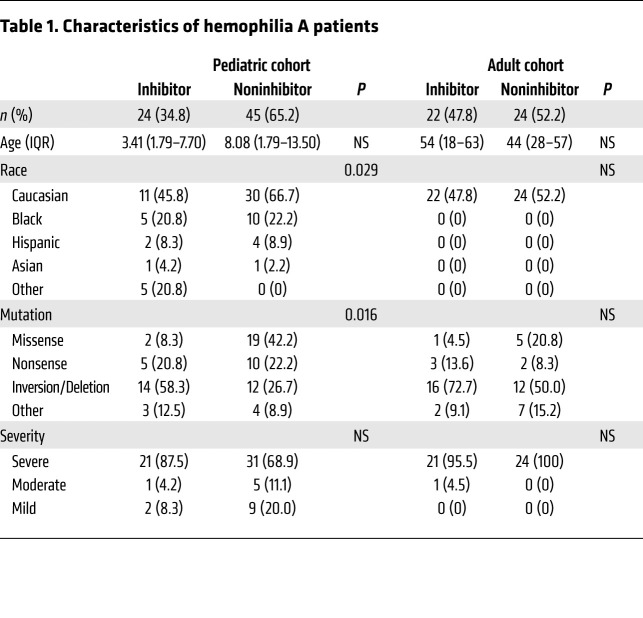
Characteristics of hemophilia A patients

## Version 1 03/02/2021

In-Press Preview

## Version 2 04/15/2021

Print issue publication
