# Supplementary material for: B cell–activating factor modulates the factor VIII immune response in hemophilia A
Source: J Clin Invest. 2021 Apr 15;131(8):e142906. doi: 10.1172/JCI142906 (PMC8262462; doi:10.1172/JCI142906)
Supplement: Supplemental data [file jci-131-142906-s162.pdf]

Supplement

Supplemental Methods

Mouse anti-human FVIII IgG ELISA

Polysorb microtiter plates (Nunc) were coated with murine IgG (Sigma-Aldrich I5381) or IgG_1 (Sigma-Aldrich M9269) standards or 1 ug/ml rFVIII overnight at 4°C. All washing steps were performed with phosphate-buffered saline (PBS, pH 7.4, Invitrogen) containing Tween (Merck). After blocking non-specific binding, samples diluted in blocking buffer were incubated overnight at 4°C. HRP-conjugated secondary goat α-mIgG-HRP (Agilent, Santa Clara, CA) or rat α-mouse IgG_1-HRP (AbD Serotec, Oxford, UK) were added for 1 hour at 37°C. Bound antibodies were detected with o-phenylenediamine dihydrochloride substrate (OPD, Millipore Sigma) in citric acid. After sufficient color development, plates were read at 492 nm. The delta optical density (DOD) for each sample was corrected for blank values.

Human anti-FVIII IgG subclass ELISA

Polysorb microtiter plates (Nunc) were coated with 1 ug/ml rFVIII overnight at 4°C. All washing steps were performed with PBS with 0.05% Tween-20. Non-specific binding was blocked with preselected buffers. Plasma samples and either anti-FVIII human IgG_1 or IgG_4 standards (provided by Dr. Voorberg) were diluted in LowCross buffer (Candor Bioscience, Wangen, Germany) and incubated overnight at 4°C. HRP-conjugated secondary mouse αhIgG_1 (Fitzgerald, Acton, MA) or IgG_4 (AbD Serotec, Oxford, UK) were added and incubated for 1 hour at 37°C. Detection was with OPD substrate in citric acid. After sufficient color development, plates were read at 492nm and OD was corrected for blank values.

Human BAFF, APRIL and soluble BCMA Multiplex ELISA

Antibody sets for human BAFF (DT124-05), BCMA (DY193), and APRIL (DY884B) were purchased from R&D Systems (Minneapolis, MN). 8-sphere strip and 4-column reservoir (SOW-01B084) were from Assay Depot/scientist.com (Solana Beach, CA). ELISA substrate (10010469) was from Cayman Chemical (Ann Arbor, MI). Assay plates (OX1263) were from E&K Scientific (Santa Clara, CA). Plates (B1202) for sample preparation and substrate were from Denville Scientific (Holliston, MA).

All ELISA reagents were prepared according to R&D protocols. Each capture Ab was diluted into PBS and coated onto strips of the column at 1 μg/ml (BAFF), 0.4 μg/ml (BCMA), and 1 μg/ml (APRIL) overnight at room temperature. Strips were washed twice and blocked with reagent diluent for 1 hour at room temperature. Samples and standards were diluted in reagent diluent and 200 ul/well were added and incubated overnight at 4ºC. Strips were washed and detection antibodies diluted in reagent diluent were added for 2 hours at room temperature and then HRP conjugate for 1 hour at room temperature. After 4 washes, the strips were transferred to a 96 well assay plate and 100 ul/well of substrate solution (Thermofisher Amplex) was incubated until sufficient color development and read on a FluoStar Omega (BMG Labtech, Ortenberg, Germany).

Supplementary Table 1. Antibodies for murine flow cytometry studies

Antibody	Tag	Clone	Manufacturer
IgM	BV510	RMM-1	Biolegend
IgM	A488	RMM-1	Biolegend
IgG	BV510	Poly4053	Biolegend
IgD	BV605	11-26C2a	Biolegend
CD23	PerCP/Cy5.5	B3B4	Biolegend
CD93	PE/Cy7	AA4.1	Biolegend
CD21	AF647	7E9	Biolegend
CD43	APC/Fire750	S11	Biolegend
CD138	PE	281-2	BD Biosciences
CD19	A488	6D5	Biolegend
TACI	BV421	8F10	Biolegend
CD19	APC EFluor780	ID3	BD Biosciences
CD95	BV605	SA367H8	Biolegend
GL7	A488	GL7	Biolegend
BAFF-R	PE	7H22-E16	Biolegend

Supplementary Figures

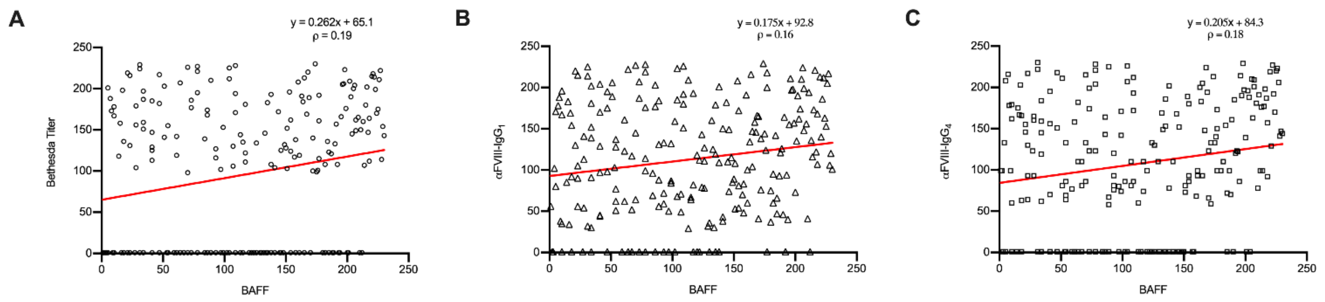

Supplementary Figure 1. Correlation plots of BAFF and FVIII inhibitor from pediatric patients. Ranked values of BAFF are plotted against ranked values of Bethesda titer (A), anti-FVIII IgG1 (B) and anti-FVIII IgG4 (C). Equations for linear correlations are listed along with spearman rank correlation coefficients.

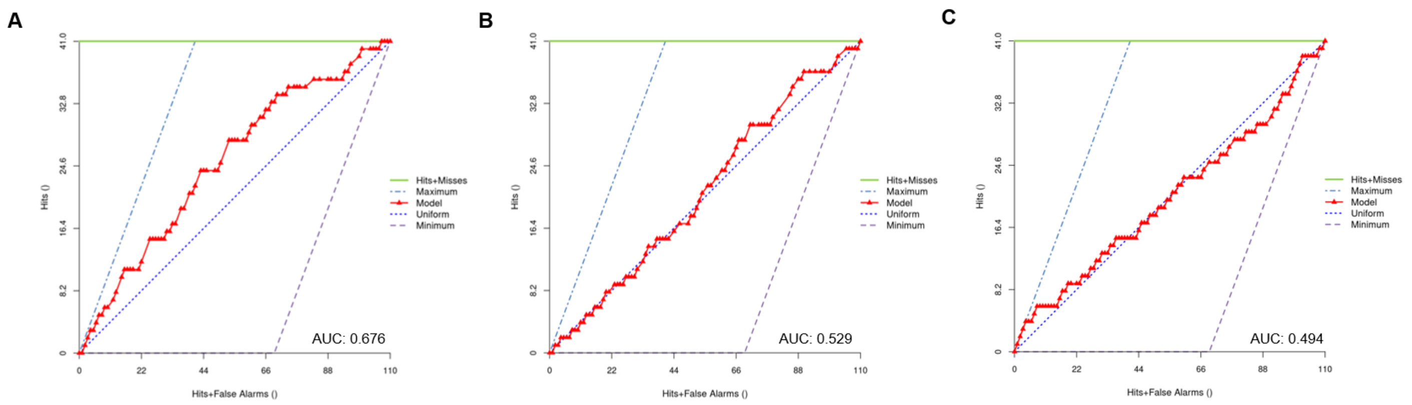

Supplementary Figure 2. Total operating characteristic curves of B cell survival cytokines in all HA patients. Total operating characteristic of A) BAFF, B) APRIL and C) BCMA in all adult and pediatric hemophilia A patients. AUC, area under curve.

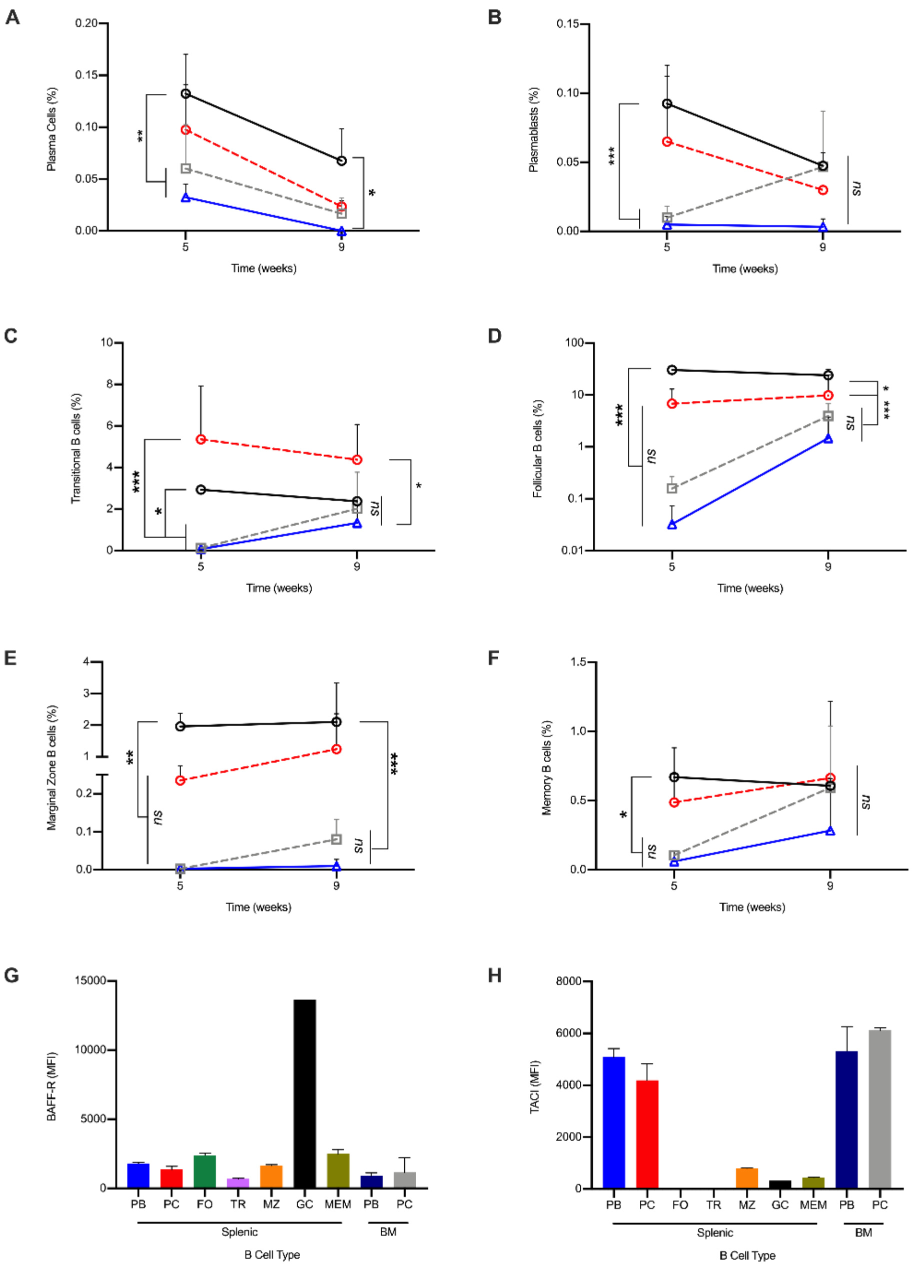

Supplementary Figure 3. B cell subsets, BAFF-R and TACI expression at early time points. Repopulation of B cell subsets in the spleens of control (o), α-mBAFF (o), α-mCD20 (☐), or α-mBAFF + mCD20 (Δ) combination therapy treated naïve HA-BALB/c mice (n=4/group), at 5 and 9 weeks following the treatment schedule shown in Figure 5A. Percent frequencies of **A)** plasma cells, **B)** plasmablasts, **C)** transitional B cells, **D)** follicular B cells, **E)** marginal zone B cells, **F)** memory B cells are indicated. Compared to controls, combination treated mice had lower FO B (30.29 vs 0.03%, $p < 0.001$), MZ B (1.96 vs 0%, $p < 0.01$), plasmablasts (0.09 vs. 0.01%, $p < 0.001$), and PCs (0.13 vs. 0.03%, $p < 0.001$) at week 5. FO and MZ B cell

depletion persisted with levels of 1.47% and 0.01% compared to 23.96% ($p < 0.01$) and 2.11% in controls ($p < 0.01$), respectively, at week 9. **G)** Median fluorescence intensity (MFI) quantification of BAFF-R expression on splenic and BM B cell subsets shows highest expression on germinal center B cells in the spleen. **H)** MFI quantification of TACI expression on splenic and BM B cell subsets shows highest expression on bone marrow plasmablasts and plasma cells followed by splenic plasmablasts and plasma cells. *ns, not significant; * p < 0.05, ** p < 0.01, *** p < 0.001 by repeated measures mixed effects ANOVA.*

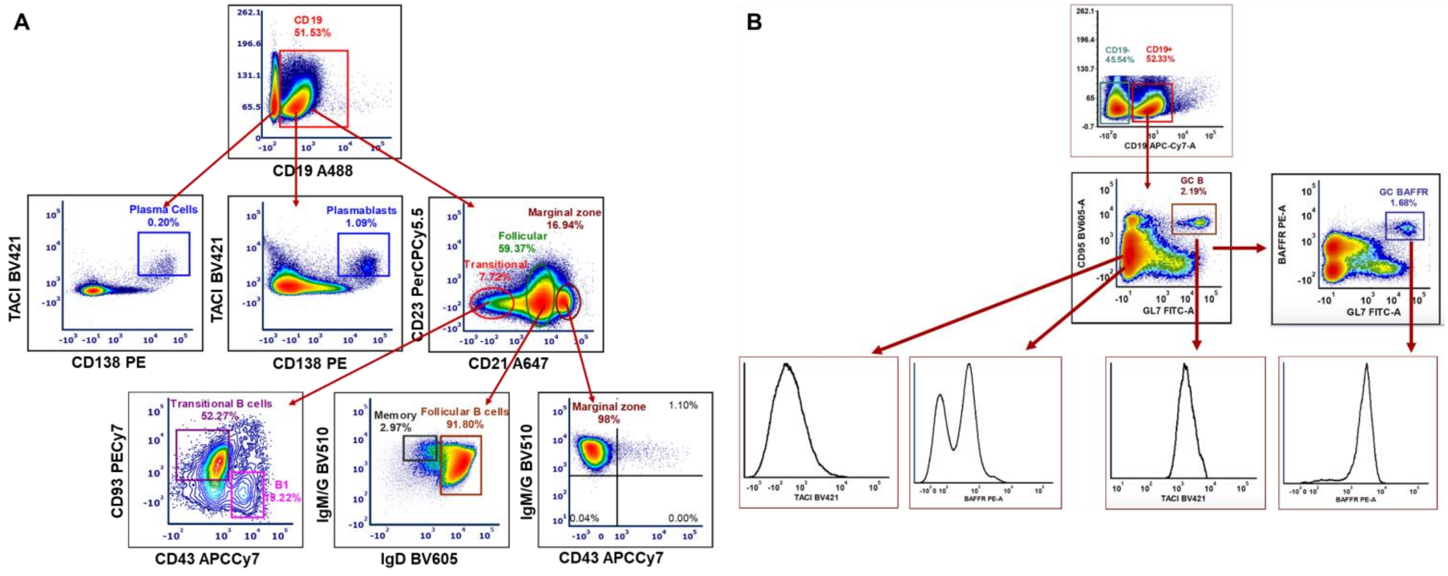

Supplementary Figure 4. Murine Flow Cytometry Gating of B cell subsets. A) Gating scheme for plasma cells, plasmablasts, and transitional, follicular, marginal zone B cells. Viable splenocyte and bone marrow cells were phenotyped into plasma cells ($CD19^{-}CD138^{+}TACI^{+}$) and plasmablasts ($CD19^{+}CD138^{+}TACI^{+}$). $CD19^{+}$ cells were further characterized by CD23 negative subsets of transitional, follicular, and marginal zone B cells based upon CD21 expression (negative, intermediate, and high, respectively). CD43 and CD93 gating was used to differentiate transitional ($CD43^{-}CD93^{+}$) and B1 ($CD43^{+}CD93^{-}$) cells. Follicular and memory B cells were classified by IgM and IgD expression and marginal zone B cells confirmed by $CD43^{-}IgM^{+}$ phenotype. **B)** Identification of germinal center B cells. HA BALB/c mice were administered with weekly 1.5 IU BDD-rFVIII i.v. combined with 50μg/mouse TLR9 agonist (CpG ODN 1826, Invivogen, San Diego, CA) for 4 weeks. $CD19^{+}$ B cells were separated by GL7-FITC and CD95-BV605 expression to identify germinal center B cells ($GL7^{+}CD95^{+}$). Intensity of BAFF-R (PE^{+}) and TACI ($BV421^{+}$) expression was compared between germinal center and follicular B cells.
